# Implementation of Privacy and Security for a Genomic Information System Based on Standards

**DOI:** 10.3390/jpm12060915

**Published:** 2022-05-31

**Authors:** Silvia Llorente, Jaime Delgado

**Affiliations:** Information Modeling and Processing (IMP) Group—DMAG (Distributed Multimedia Applications Group), Computer Architecture Department, Universitat Politècnica de Catalunya · BarcelonaTech, 08034 Barcelona, Spain

**Keywords:** genomics, privacy, security, modular architecture, GIPAMS, standards

## Abstract

Genomic information is a very sensitive type of digital information as it not only applies to a person, but also to close relatives. Therefore, privacy provision is key to protecting genomic information from unauthorized access. It is worth noting that most of the current genomic information formats do not provide specific mechanisms by which to secure the stored information. In order to solve, among other things, the privacy provision issue, we proposed the GIPAMS (Genomic Information Protection And Management System) modular architecture, which is based on the use of standards such as ISO/IEC 23092 and a few GA4GH (Global Alliance for Genomics and Health) initiatives. Some of the GIPAMS modules have already been implemented, mainly based on ISO/IEC 23092 features, and we are conducting work on the complete version of the architecture, and other standards are also considered. One of the objectives of GIPAMS is to enable the management of different formats of genomic information in a unique and interoperable way, providing privacy and security for formats that do not currently support them.

## 1. Introduction

Genomic information is a very sensitive type of digital information. If it is leaked, it becomes public “forever”. However, this is not the worst of the potential consequences; if some genomic information is leaked, not only will one person’s information be revealed, but her close relatives’ information is also impacted. So, it is key to define mechanisms for providing privacy and security related to genomic information.

Genomic information is currently stored in different file formats and locations, making it difficult for researchers, and, in general, health professionals to access it. Moreover, file sizes may be up to several hundreds of GB, complicating its transmission and manipulation. Finally, researchers need to follow strict authorization procedures to access some genomic studies, hindering possible research advances due to the complexity of such an authorization process.

In order to solve some of these issues, we present our proposal for a secure modular system in this paper, called GIPAMS (Genomic Information Protection And Management System) [1]. With this system, we attempt to provide a solution to the problem of privacy protection of genomic information. It implements mechanisms for providing privacy as well as secure storage, search and access to genomic information. We already described a preliminary version of this system in [1], but it is still under improvement, as we explain in this paper. Although the proposed system architecture is standards-agnostic, most of the modules implemented in this version are based on the ISO/IEC 23092 standard, also known as MPEG-G (Genomic Information Representation) [2,3], as we further describe in the following sections.

Nevertheless, ISO/IEC 23092 is not the only way of representing genomic information. The Global Alliance for Genomics and Health (GA4GH) [4] also defines protected genomic information representation formats, such as Crypt4GH [5], and ways of authorizing and authenticating access to genomic information, such as GA4GH Passports [6,7] and Authentication and Authorization Infrastructure (AAI) [8,9]. Moreover, they embrace some of the most relevant genomic file formats such as SAM/BAM [10] or CRAM [11] for the representation and storage of genomic information. Based on this, we also describe how GA4GH standards can help us in improving GIPAMS.

The aim of the work presented in this paper is to describe how genomic information can be managed in a secure way using a modular architecture (GIPAMS [1]) based on standardized information formats, specifically ISO/IEC 23092 [2,3]. Moreover, we explain some implementation details (programming languages, tools, etc.) of the GIPAMS architecture together with improvements we plan to apply to the current version. These improvements include, among other things, the use of other secure standardized formats, such as Crypt4GH [5], or authorization technologies such as AAI [8,9] and GA4GH Passports [6,7], all coming from GA4GH [4]. To do so, we propose the “duplication” of some modules of the architecture in order to support the different file formats and protection and authorization mechanisms. Other possible improvement is the implementation of a federation of GIPAMS to facilitate communication and interoperability between different organizations in charge of genomic information storage and management.

In the rest of this section, we briefly describe ISO/IEC 23092, as it will be further developed in Section 1.1 and Section 2.2. Moreover, we describe some GA4GH mechanisms for the provision of privacy and security for genomic information.

### 1.1. ISO/IEC 23092 (Genomic Information Representation)

ISO/IEC 23092 Information Technology - Genomic Information Representation [2,3] is a standard devoted to the representation of compressed genomic information including appropriate security and privacy features [12,13,14] since its inception following a Security and Privacy by Design approach [15]. It is composed of six parts, most of them already published. Some of them are even under revision for new editions [16,17,18,19,20], and one of them is still under development [21]. Part 1 is devoted to the Transport and Storage of Genomic Information. Part 2 describes the Coding of Genomic Information. Part 3 is more relevant for this work, as it defines Genomic Information Metadata and Application Programming Interfaces (APIs). Part 4 specifies the Reference Software. Part 5 addresses Conformance, and Part 6 defines the coding of genomic annotations.

The ISO/IEC 23092-3 is devoted to protection [18]. Amongst other security features, it defines the selective encryption of sequencing data and metadata, and enforces privacy with the definition of privacy rules that can also be applied to genomic data and associated metadata. In addition to privacy rules and encryption, digital signatures can be applied to the information to assure integrity by preventing tampering. However, signatures are not the only mechanism to prevent the tampering of genomic data, as fingerprinting has also been considered [22].

These protection mechanisms can be applied at different levels inside ISO/IEC 23092 conformant files, providing high granularity when securing genomic data [12,13]. Each identifiable portion of the coded file can be encrypted to protect confidentiality or signed to protect integrity. In order to provide access control to the protected information, encryption and privacy rules have to be combined, ensuring that only authorized users can perform an action on the information. 

ISO/IEC 23092 provides a syntax expressed in XML language [23], to support different strategies for encrypting and signing genomic information. For instance, we could define encryption and access control with privacy rules for data and only signature for metadata. Another scenario could be the encryption and application of privacy rules associated with just metadata, leaving out genomic information. Possible protection strategies also provide support for key retrieval, key derivation, key wrappers and, as already mentioned, signatures [18]. Some of these ideas are described in more detail in [24]. In Section 2, we describe ISO/IEC 23092 standard features and how we used them to design and implement GIPAMS.

### 1.2. Global Alliance for Genomics and Health (GA4GH)

The Global Alliance for Genomics and Health (GA4GH) [4] defines itself as “a policy-framing and technical standards-setting organization, seeking to enable responsible genomic data sharing within a human rights framework”.

As mentioned before, there are several initiatives within GA4GH that manage security, privacy, authentication and the authorization of access to genomic content, at different levels, such as Passports [6,7], Authentication and Authorization Infrastructure (AAI) [8,9] and Crypt4GH [5]. GA4GH also defines communication protocols to interchange genomic information, such as the htsget API [25,26]. htsget defines a data retrieval application programming interface (API) based on well-known standards and recommendations such as HTTP(S), JSON [27] and OAuth 2.0 [28]. It supports the retrieval of data for different genomic data formats, such as SAM/BAM [10] or CRAM [11]. 

We briefly describe some of these initiatives in the following Section.

#### 1.2.1. GA4GH Passports

**Figure 1:**
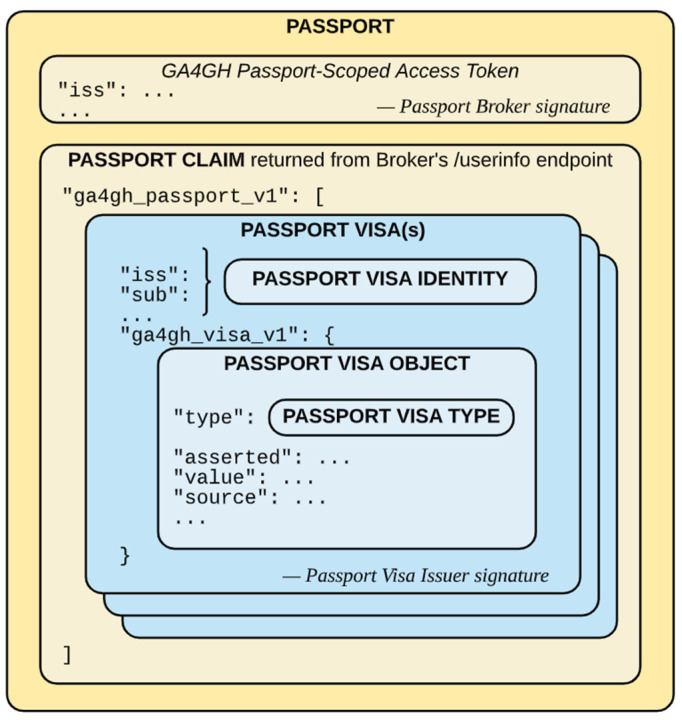
The composition of objects and tokens within a Passport. Source [7].

GA4GH Passports [6,7] define a mechanism by which to determine whether a researcher can access some genomic information based on the data contained in her passport. The structure of a GA4GH Passport is sketched in Figure 1. Objects and tokens are grouped together as presented. There are two separate sections inside the passport, one for the Access Token and the other for the Passport Claim, where different Passport Visas can be found. Each Passport Visa can be issued by a different organization, whose signature is included to provide integrity and authentication to the issued visa. 

**Figure 2:**
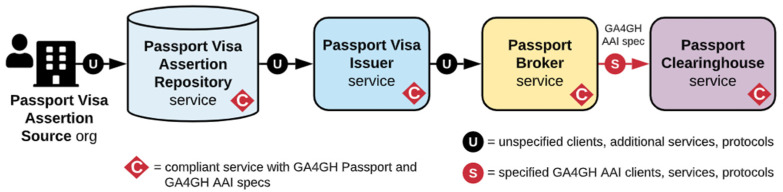
Basic flow of data from Passport Visa Assertion Source to Passport Clearinghouse. Source [7].

The basic flow of data from the Passport Visa Assertion Source to Passport Clearinghouse Service is shown in Figure 2. As it can be seen, there are different services involved in the process. Some of the steps are completely defined by GA4GH in [7], such as the one between Passport Broker and Passport Clearinghouse services. Other services just need to be compliant, such as the ones involved with Passport Visa, and others remain unspecified, such as the communication between services until reaching the Passport Broker.

Some limitations of the first version of GA4GH Passport led to the development of version 1.2, which will allow for the separation of Passport visas from other personal information, and 2.0, which attempts to solve more sophisticated security issues in the token interchange process.

#### 1.2.2. GA4GH Authorization and Authentication Infrastructure (AAI)

GA4GH Authorization and Authentication Infrastructure (AAI) [8,9] defines mechanisms for authenticating an individual and authorizing access to a dataset, independent of being genomic or not, as GA4GH AAI claims to be “domain agnostic”. It makes use of the OpenID Connect standard [29] to introduce the concept of the “access token”. 

In particular, AAI profiles the OpenID Connect protocol to provide a federated authentication and authorization infrastructure between genomics institutions in order to facilitate interoperability, especially when, but not limited to, sharing restricted datasets.

The relationship between GA4GH Passports and AAI is that AAI provides a method for identifying users and transporting claims related to them, whilst Passports provide the data format for converting user claims into permissions associated with datasets, user roles, resources and more. 

In this way, an AAI access token can be included in the Passport to transport a researcher’s digital identity and permissions. Then, these permissions can be mapped to the specific organizations, tools and environments, favoring federation handling, as it can manage different organizations, tools and environments. 

#### 1.2.3. Crypt4GH File Format

Crypt4GH [5] is the file format defined by GA4GH [4] used to encrypt genomic files to provide them with confidentiality, integrity and authentication. To do so, the use of several encryption keys is defined, both symmetric and asymmetric, which are used to protect both header packets and genomic data. 

**Figure 3:**
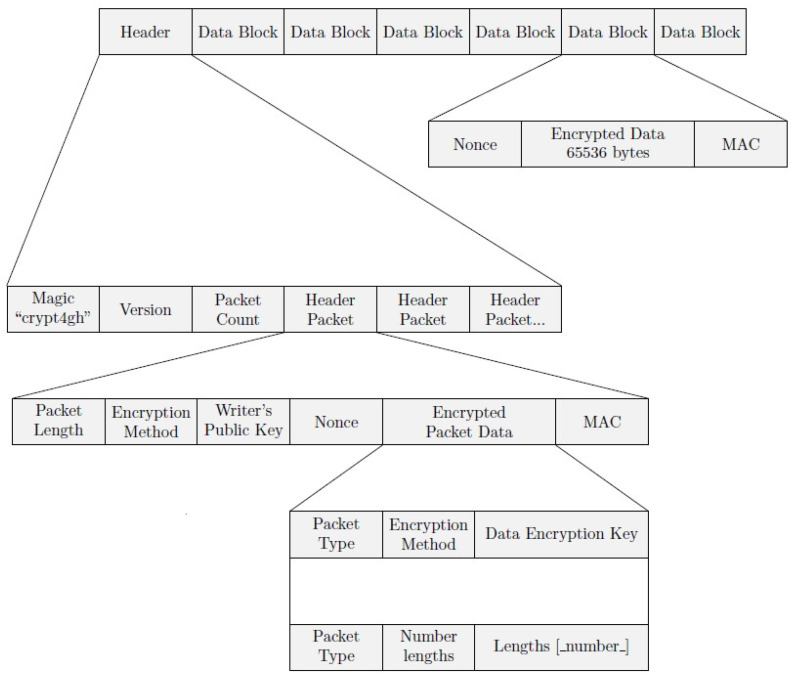
Crypt4GH file structure. Source [5].

Figure 3 shows the file structure. It can be seen that the first level of this structure consists of Header and Data Blocks. Inside the Header, we can find Header Packets containing encryption information, which are basically the keys and encryption methods needed to decrypt the genomic data for the different users with access to the file. A combination of asymmetric reader and writer keys is used to obtain the symmetric key used for encrypting data blocks. Both header packets and data blocks contain a Message Authentication Code (MAC), to ensure its authentication.

To process the file, the reader first has to read the Header, checking that the magic number and version provided match the expected values. Then, she should attempt to decode all header packets, ignoring those whose MAC cannot be verified, as this means that this specific header packet was not intended for this reader. If no header packet has been verified after processing all of them, an error has to be reported. If there is more than one header packet verified, the reader must store all the keys decoded in order to process the corresponding data blocks.

The data blocks decoding process involves the authentication and decryption of the segment(s) enclosing the data range required by the reader, expressed by two numbers, P and Q, where P < Q. 

To decrypt the required data range, the initial segment, including the Nonce and the MAC, is read. After that, an authentication tag is calculated over the cipher-text and compared with the MAC read from the file. The cipher-text is authenticated if and only if both tags match. If there is more than one key for decrypting data in the header, each one has to be checked until one authenticates the segment or no keys are left. In case no key authenticates the cipher-text, an error should be reported. 

To effectively decrypt the cipher-text, the key authenticating the MAC and the Nonce are used, returning the corresponding plain text. The process is repeated for the subsequent segments, until the segment containing position Q is reached. 

Ref. [5] defines the encryption and authentication methods accepted together with some considerations on how to improve the decryption process after keys are decoded from the header packets.

## 2. Materials and Methods

This section describes in detail the development and set-up of the Genomic Information Protection And Management System (GIPAMS) [1]. Some of the proposed modules have an initial implementation that can be accessed at [30]. It is worth noting that this is not an outline of the complete implementation of the system, as it is currently under development as further explained in the following subsections. Moreover, we also describe how GIPAMS follows some of ISO/IEC 23092 features. 

### 2.1. Genomic Information and Protection Management System (GIPAMS)

**Figure 4:**
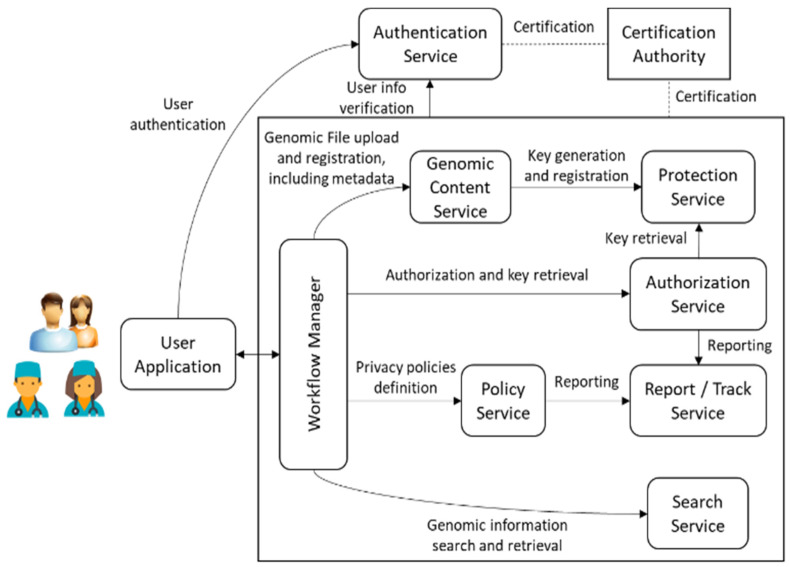
GIPAMS architecture.

The Genomic Information Protection And Management System, GIPAMS, is an evolution of our original Multimedia Information Protection And Management System, MIPAMS [31]. It transitioned from managing multimedia content to genomic content, but the underlying concepts remain, i.e., providing a secure standards-based modular architecture for managing the information and its associated metadata. To achieve this, we used the features defined in the different parts of the ISO/IEC 23092 GIPAMS architecture and structure, as depicted in Figure 4. 

We already performed a partial pilot implementation [1] of the architecture mainly based on ISO/IEC 23092 features. Nevertheless, this is the expected final complete picture, where other standardization initiatives may be considered, as further described in Section 4.1. The functionality of the different modules is as follows:

- User Application (UA): Access point to the whole system. It sends all requests to the Workflow Manager, which redirects to the corresponding module based on the action requested by the user. An access token is required, which is provided by the Authentication Service. Communication between this application and the rest of the architecture is performed through a secure channel. It is currently implemented as a web application but it could be a desktop application or even a mobile application.
- Workflow Manager (WM): Intermediate module that acts as a unique entry point to the system to facilitate interactions with the other modules, making them transparent to the final user. Before redirecting to the module in charge of an operation coming from the User Application, it checks if this operation is authorized using the information inside the access token.
- Authentication Service (ATS): Server in charge of user identification. It provides authentication features using OAuth 2.0 [28] and JSON Web Tokens [32]. Its implementation is currently based on Keycloak [33], although other providers may be considered, such as FusionAuth [34] or Gluu [35].
- Genomic Content Service (GCS): Module in charge of genomic archives management, both in reading and writing operations. In case genomic data has to be protected (encrypted or signed), it may connect with the Protection Service to obtain the required keys. It also manages metadata storage.
- Authorization Service (AS): Module which validates authorization rules. It is currently based on WSO2 Balana [36]. Authorization requests are usually sent from the Workflow Manager responding to user actions, but other modules may also interact with it to request the authorization of internal operations.
- Search Service (SS): Module which performs searches over genomic information (especially metadata). In order to provide extra filtering features, it uses a relational database where metadata fields are stored. It must be checked by means of authorization rules so that the returned results can be seen by the user requesting them.
- Policy Service (PS): Module in charge of the creation of the authorization rules. In the current version of this module, which makes use of eXtensible Access Control Markup Language (XACML) [37], they are organized into XACML policies and rules.
- Protection Service (PTS): Module which creates protection information metadata associated with genomic information. It applies the defined mechanisms (i.e., encryption, signature, etc.) to the corresponding genomic data or metadata.
- Report/Track Service (RTS): Module in charge of reporting the operations implemented in the system, especially those not authorized. It helps in keeping track of illegal/unusual operations that may indicate an attempt to attack the system.
- Certification Authority (CA): This is not a real module of the system, but something required for its proper functioning. It provides the certificates needed to establish secure connections between the different system components.

### 2.2. ISO/IEC 23092 Relationship with GIPAMS

As already mentioned throughout this paper, ISO/IEC 23092 was adopted as a starting point for the implementation of GIPAMS (Genomic Information Protection And Management System) architecture as it provides different features that can be separated into interconnected modules to provide the complete picture. Some of these features are outlined below.

- Hierarchical organization of the genomic information thanks to the file structure defined in the standard [16].
- Compression of genomic information, by means of standardized compression algorithms [18].
- Metadata at different levels of the hierarchy using the corresponding metadata box at the information level it applies, such as file, dataset group, dataset or access unit [18].
- Privacy rules used for access control on both genomic data and metadata, are stored in the corresponding protection box at the information level and apply to dataset groups or datasets [18]. This structure is described in Section 2.3.
- Encryption and protection mechanisms both for genomic data and metadata, stored in the corresponding protection box at the information level it applies to, with dataset groups, datasets or access units [18].
- Integrity by means of digital signatures, which can be applied to genomic data and metadata, stored in the corresponding protection box at the information level it applies to, including dataset groups, datasets or access units [18].

### 2.3. ISO/IEC 23092 File Structure

**Figure 5:**
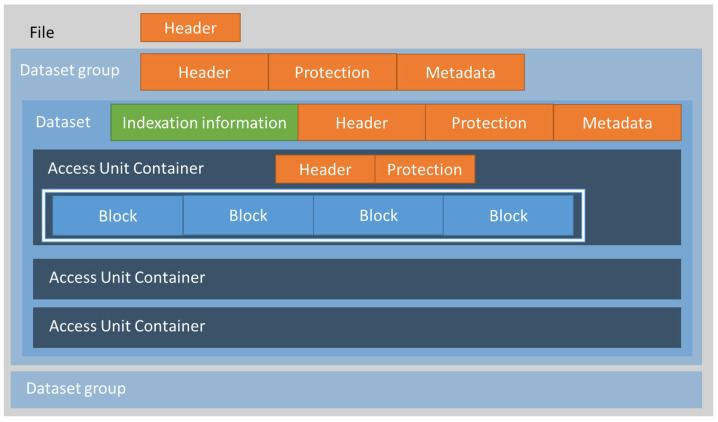
ISO/IEC 23092 basic file structure.

In order to support the security strategies proposed in ISO/IEC 23092, a hierarchical file format was defined, as shown in Figure 5. This file format combines protection and metadata elements with the genomic data. It is worth noting the security and privacy using the design approach taken into account in the definition of this file format.

Figure 5 shows the standardized format of an ISO/IEC 23092 file when working in an Access Unit Container (AUC) mode. When using AUC, genomic information is stored as access units, which are sets of coded genomic information that can be independently accessed and inspected. There is another mode, called the Descriptor Stream Container (DSC), where genomic information is organized in a different way, specifically, as descriptor streams. Both modes may contain the same base genomic information, although the difference lies in how it is organized, which depends on how and when genomic information has to be generated, processed and accessed. We will focus on the AUC mode for the description of the features presented.

The file is structured in hierarchical boxes, with the File as the root element, which has a header element, including basic file information. Inside the file structure, we can find Dataset Groups, which also have an associated header as well as metadata and protection information. The Dataset Group may contain one or more Datasets, which, in turn, may contain information boxes or other container boxes. The last level of the hierarchy may be organized in Access Units (AUC mode), as shown in Figure 5, or in Descriptor Streams (DSC mode), depending on how genomic information needs to be accessed. In the end, genomic information is stored in Blocks, regardless of the use of Access Units or Descriptor Streams. Figure 5 shows an example of a Dataset Group containing several Datasets, as well as support for protection and metadata. The indexation information appearing at the Dataset level supports direct access to different parts of the genomic information.

The advantages of the ISO/IEC 23092 file structure include the hierarchical organization and the fact that metadata and protection information are stored together with the genomic information itself, as described in Section 2.4 and Section 2.5. Moreover, other advantages include the existence of an API for accessing information and the availability of authorization policies and rules also included inside the file. Finally, indexes can be defined to provide direct access to specific regions of the genomic information. The main current disadvantage is that it is not widely accepted for the moment, as there are well established genomic information formats used both in genomic research and clinical practice.

### 2.4. ISO/IEC 23092 Metadata

The hierarchy defined in ISO/IEC 23092 attempts to represent the metadata structures for different organizations, such as the European Genome-Phenome Archive (EGA) [38] and the National Center for Biotechnology Information (NCBI) [39], among others. The Dataset Group represents concepts such as Study in EGA or BioProject in NCBI. Analogously, each dataset corresponds to a dataset in EGA or BioSample in NCBI.

ISO/IEC 23092 part 3 [18] stores metadata in the information boxes Dataset Group Metadata (dgmd) and Dataset Metadata (dtmd) using Extensible Markup Language (XML) [23]. We created some mappings in order to check that the metadata coming from EGA and NCBI are compatible with the developments required for GIPAMS architecture. The results of this mapping were diverse for different reasons, briefly outlined in the following section. 

EGA uses a similar structure, so the mapping is direct, as represented in Table 1. For NCBI, the mapping was not as easy to achieve, as there are some differences. For example, the Abstract field is not present in NCBI metadata and their Type is not identical in both cases. This mapping is represented in Table 2.

On the other hand, ISO/IEC 23092 provides a mechanism by which to store additional information inside metadata fields. To test this mechanism, we defined some extensions for the NCBI metadata, represented in Table 3 and briefly described next. The field StudyDesign indicates an epidemiological or omics research design context in which a sample was used. BodySite stores information about the type of tissue the sample was taken from. AnalyteType represents the biological specimen sampled from a subject (e.g., DNA from blood). The IsTumor indicates whether the sample corresponds to a tumor or not. These mappings have their corresponding XML representation according to ISO/IEC 23092 part 3 [18].

### 2.5. ISO/IEC 23092 Privacy Rules

In current version of ISO/IEC 23092, privacy rules are expressed in the eXtensible Access Control Markup Language (XACML) standard [37], from OASIS [40]. XACML defines several information structures in order to support authorization mechanisms. 

Regarding the definition of policies and rules, it describes the elements PolicySet, Policy and Rule. A PolicySet may have some Policy elements, which, in turn, may contain some Rule elements. XACML defines some algorithms to combine policies and rules when authorizing some action. 

On the other hand, in order to ask for authorization, the XACML Request concept is required. These requests include the attributes that are sent to the XACML authorization mechanism, which attempts to match with the existing policies and rules in order to return an authorization decision.

Inside XACML rules, different information elements can be defined, as presented in [12,13,14]:

1. Who is able to access to the genomic information (user roles or individuals);
2. Wat information can be accessed (the complete file, a chromosome, etc.);
3. When it can be accessed;
4. With which purpose (genetic analysis, anonymized study, etc.) it can be accessed;
5. Whether the data provider has to be informed when information is accessed; and
6. Which specific permission is provided.

In our approach, the privacy rules are represented using XACML and the possible actions are defined in the ISO/IEC 23092 Application Programming Interface (API) [18]. We convey privacy rules only at the Dataset Group and the Dataset levels, as there are no specific actions for Access Unit level. An XACML policy element is included in the ISO/IEC 23092 protection XML schema. In this way, each protection element inside the file may have its corresponding privacy policy, which, in turn, may have several privacy rules, controlling access to different parts of the file.

The methods defined in the API also use other attributes. For example, in GetDataBySimpleFilter, the user can select whether multiple alignments [41] should be considered in the returned result. Filtering can be relevant to the privacy rules (most notably when used to delimit the returned region of the genome); therefore, in our proposal, this should also be included in the request. This translates, for example, into an attribute with its ID equal to presence_of_multiple_alignments. 

**Figure 6:**
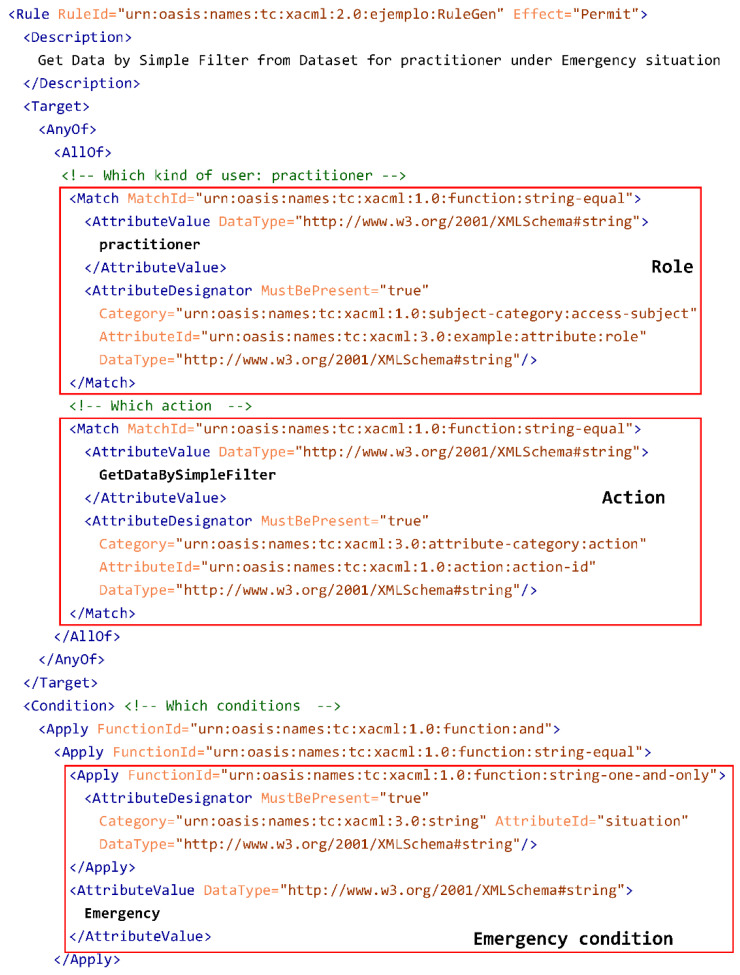

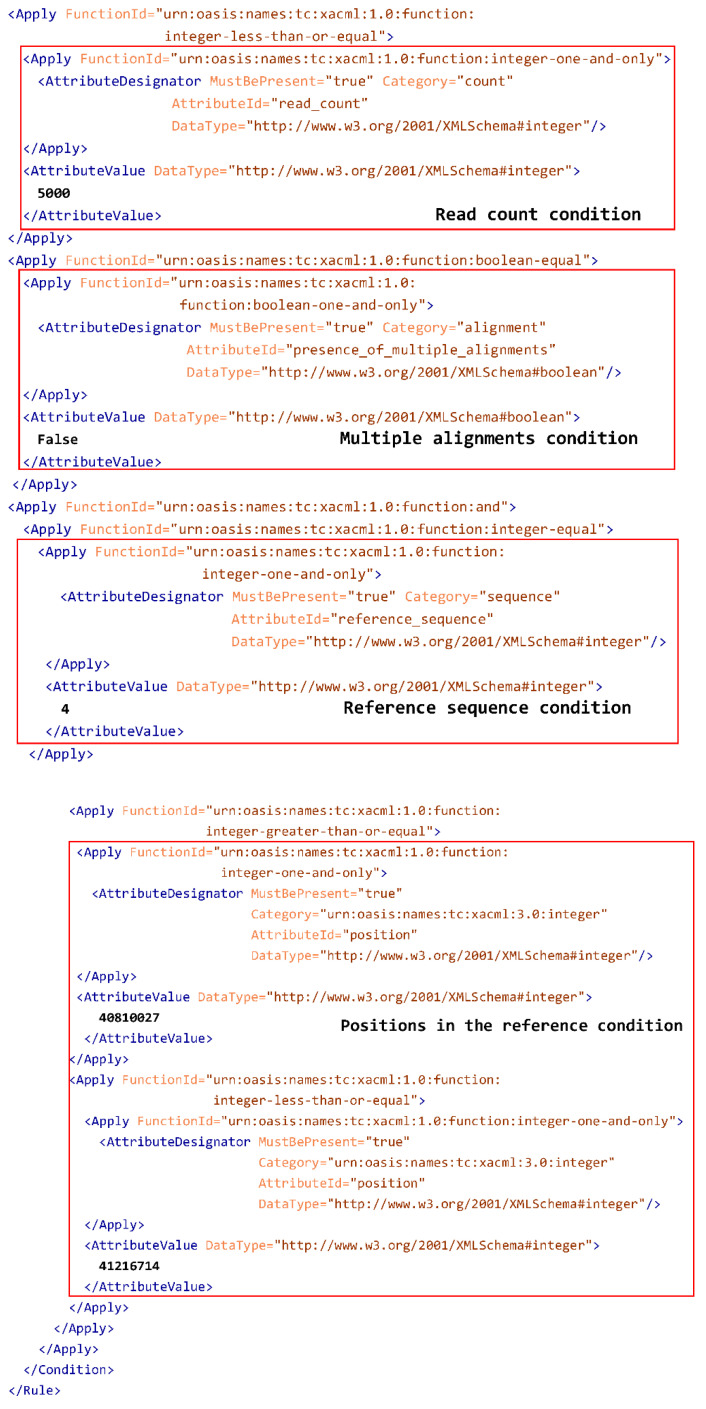
XACML Genomic rule example.

The rule shown in Figure 6 allows for the execution of the operation GetDataBySimpleFilter by the role practitioner under some conditions intended to protect regions helping to identify Alzheimer’s disease predisposition (it is not an exhaustive list, as some conditions are missing):

- Under an emergency situation.
- For a read count of 5000.
- Without multiple alignments.
- For reference sequence equal to 4, considering a range between 40,810,027 and 41,216,714, with both extremes included.

For the particular case of ISO/IEC 23092 file elements, the definition of the resource is not required as the rule applies to the Dataset or Dataset Group where it is contained. So, the ISO/IEC 23092 file structure already provides the relationship between the rule and the data to which it refers (either a Dataset Group or a Dataset). Therefore, there is no need to link both in the rule, as the resource is implicitly associated with the policy. 

Figure 6 shows the rule constructed to represent this information. First of all, it represents the role, practitioner, indicating that it is an attribute called role, defined in XACML version 3 [37], which is of category access-subject (defined in XACML version 1, as indicated by urn:oasis:names:tc:xacml:1.0:subject-category:access-subject). Then, the permitted action, GetDataBySimpleFilter, is defined as an action-id attribute of category action. Then, the Emergency condition is defined, using the attribute situation, specific for this rule. The rest of the conditions, the read count, the presence of multiple alignments, reference and positions in the reference, are also defined for this rule, not belonging to any XACML standardized category. It is worth noting the flexibility provided by XACML, as it helps to define the required attributes and categories for a specific use case such as ours.

**Figure 7:**
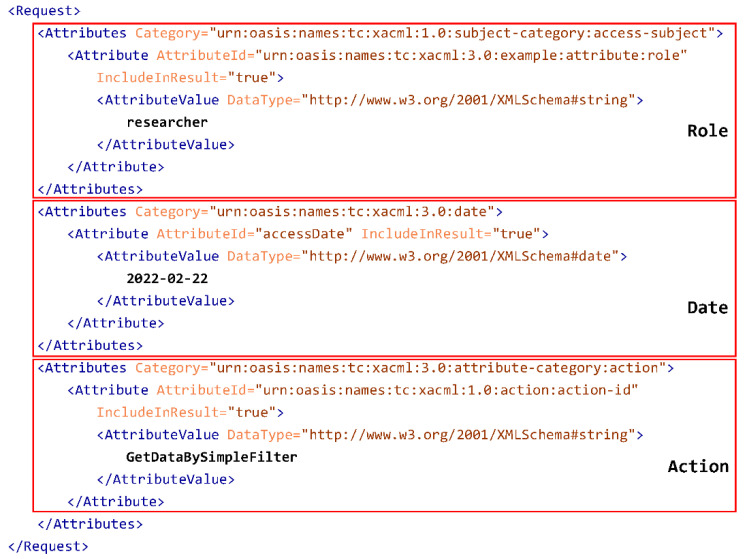
XACML Genomic request example.

Figure 7 shows an example of an XACML request considering some attributes such as role, date and action. The authorization result of this XACML request according to the XACML rule is Deny, as the role is not the same as the one defined in the rule shown in Figure 6. 

On the other hand, it is also possible to protect the privacy of other parts of the ISO/IEC 23092 files, such as metadata. In this case, the rule indicates the operation that can be performed over metadata (contained in the Dataset or the Dataset Group). Then, the rule(s) should be checked before a user or role performs any operation over metadata.

### 2.6. Authorization Based on ISO/IEC 23092 Hierarchy

The Application Programming Interface (API) methods defined for Dataset Group and Dataset in ISO/IEC 23092 are very similar. For instance, a user can request data with the GetDataBySimpleFilter method to retrieve information either from a Dataset Group or a Dataset. In the end, they are different methods, as each apply to a different data structure, but the privacy rule may have the same syntax. This may lead to multiple rules for the same method, applied to different levels of the hierarchy.

However, we can expect certain homogeneity when providing permissions. For example, in a Dataset Group for research purposes on Alzheimer’s, we can expect all requests to Alzheimer’s related regions of the genome to be granted. To simplify the privacy rules that need to be defined, we would ideally use a mechanism that delegates the permission for the dataset(s) to the container dataset group. Nevertheless, this mechanism should not impede on one dataset so as to diverge from the rules applied to the container dataset group. In other words, the permission for a dataset must be able to rely on a default dataset group-wide policy, but the dataset group-wide policy should not hide specificities of the dataset.

To manage this situation, we propose the following two algorithms: one for asking for access to a specific dataset and another one for asking for access to the complete dataset group. They are described in detail in the rest of the section, but they were originally introduced in Daniel Naro’s PhD Thesis [24].

**Figure 8:**
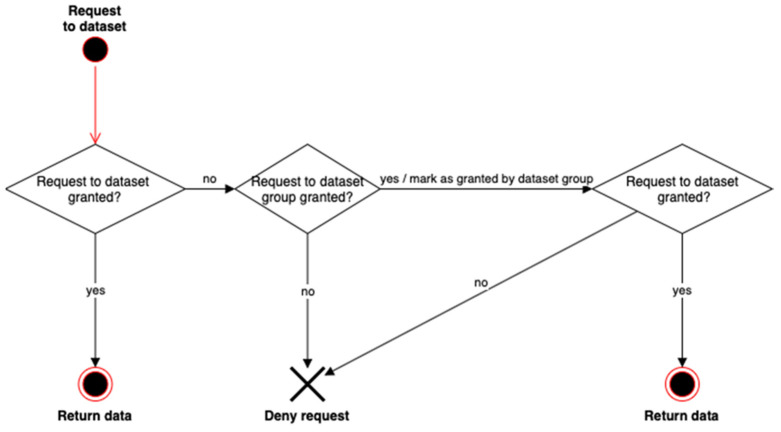
Privacy rules inheritance. Granting access to dataset from dataset group.

Figure 8 represents a case where the user makes a data request of information contained in a Dataset. In this case, only dataset-specific rules are checked. If permission is granted, then the corresponding data is returned. If not, the algorithm checks whether the equivalent request to the dataset group is granted, that is, the rules in the upper level are checked. If so, an additional attribute granted by the dataset group is added to the original request for the dataset.

**Figure 9:**
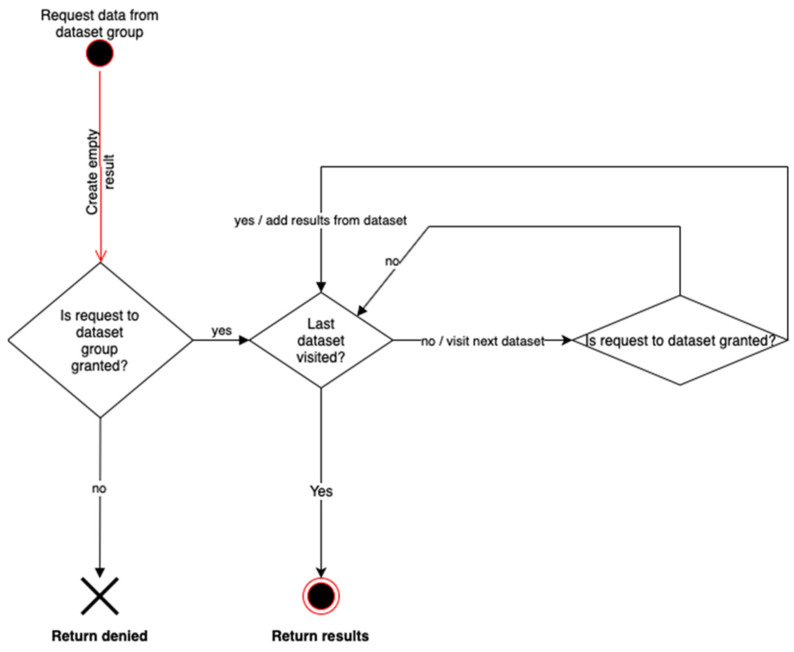
Privacy rules combining data from multiple structures. Check that dataset group rules are compatible with dataset rules.

On the other hand, we want to prevent those denied access from accessing information. The process is shown in Figure 9, where it can be seen that, if access to the dataset group is granted, access to the specific datasets should be reviewed one by one to ensure that there are no different access rules for any of them. So, we navigate in the structure, going down in the hierarchy. In that case, specific rules for Datasets are checked, blocking the return of the data associated with this Dataset, if it is not granted by the corresponding rules. The granted results have to be stored during the process, in order to return the data coming from the granted Datasets to the user.

It is worth noting that, from the user perspective, it is required to build a, XACML Request containing some attributes that will be checked against the rules contained in the genomic file. As already explained in Section 2.5, these rules may be inside a dataset or dataset group, depending on the requested data and the authorization process required (going up or going down for the processing of the rules), as shown in Figure 8 and Figure 9.

## 3. Results

This section describes the research findings of this work. Some preliminary results were already sketched in [1]. Its source code can be found in [30]. 

Section 3.1 describes our implementation of the hierarchical ISO/IEC 23092 file structure to manipulate it in an easier way. It is worth noting that an ISO/IEC 23092 file size could reach up to several hundreds of GB, so, maintaining it in memory to perform different operations, such as accessing encrypted information, is a “hard” task for any program. In this way, splitting the file into several smaller files facilitates the creation and modification of the internal structure. Once the complete structure is created and protected, one can generate the MPEG-G file following the ISO/IEC 23092 structure.

Section 3.2 describes how the different modules are implemented, including details on programming language and architecture.

Some specific details of the implementation process are also described in the rest of this section.

### 3.1. File Structure Implementation

**Figure 10:**
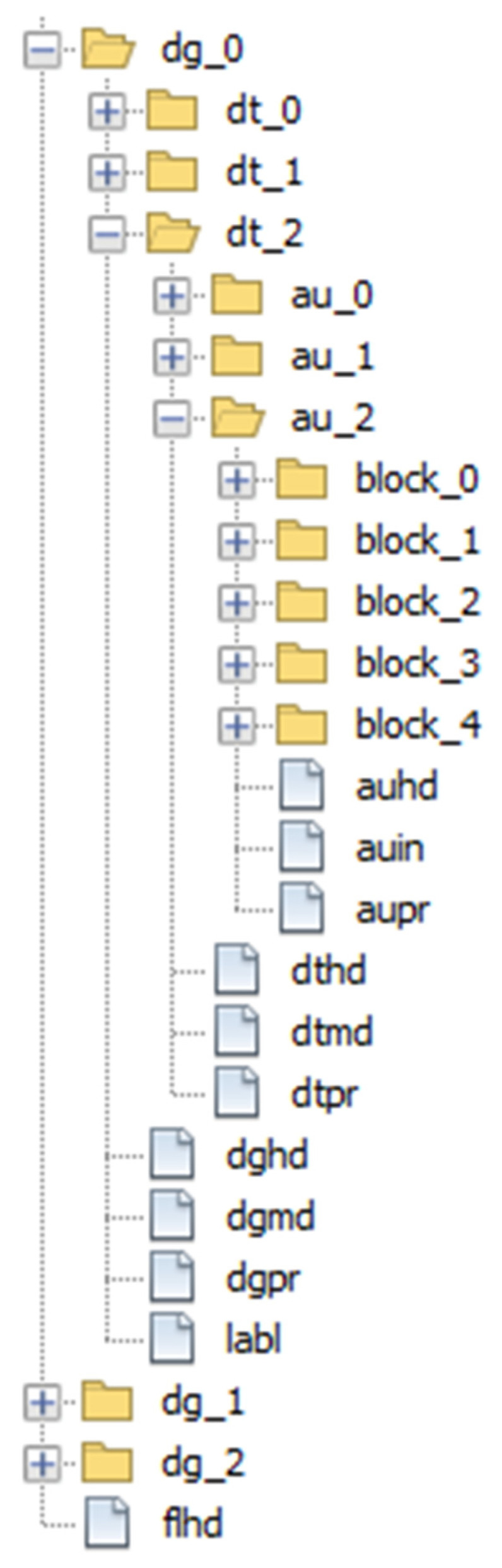
File structure example.

As explained in Section 2.3, ISO/IEC 23092 files are structured in hierarchical boxes forming a single file. As already mentioned, the size of this file could be up to several hundreds of GB. To avoid having to deal with such large files and for the sake of testing, we used an alternative approach to simulate this structure, which consists of using the file system (folders and files) to represent the box hierarchy, as shown in Figure 10. In the end, the storage space used by our approach could be even greater than the ISO/IEC 23092 file. The point here is that we can access and manipulate all the information in an easier way, navigating through the folders and files.

In this approach, every box is represented by a directory, which may have several files inside containing the header, metadata or protection information, depending on the elements present in a specific box according to the corresponding hierarchy level. Moreover, some subdirectories representing the inner boxes, that is, datasets inside dataset groups and so on, are also created. Each subdirectory may contain files associated with its hierarchy level. It is worth noting that some elements are optional, such as metadata or protection, so, they may not be present. So, in Figure 10, dataset groups are contained in dg_X folders, datasets are contained in dt_X folders, Access Units are contained in au_X folders and block_X folders contain the different Blocks inside an Access Unit. 

Header files present in the boxes follow the pattern xxhd, where xx may be au for access units, dt for datasets, dg for dataset groups and fl for the complete file. Protection files follow a similar naming pattern, but using xxpr. Metadata files use the naming convention xxmd. The other files appearing in Figure 10 are labl, which represents the label element at the dataset level and auin, which stands for access unit information. The complete file structure can be found in [16].

The information files still need to be manipulated at the bit level, so we developed a Python script [30] that can generate this whole directory structure and create the information files for each hierarchy level using valid data. The script can also integrate real metadata and protection policies into the files, using the data provided by users in the form of XML files. Once the file system structure is created, the complete ISO/IEC 23092 file can be constructed from the directories and files stored in disk, in case it needs to be shared with some other researcher or organization. 

### 3.2. Modules Implementation

GIPAMS modules [1] use the Java programming language [42] by implementing J2EE (Java 2 Platform Enterprise Edition) [43] compliant web applications. This is the base for User Application (UA), Workflow Manager (WM), Genomic Content Service (GCS), Authorization Service (AS) and Search Service (SS) modules. Some implementation details are provided in the following.

Keycloack [33] is used as the authentication provider. It is an open source software used by the UA to obtain a JSON Web Token (JWT) [32]. Then, this JWT is used to authenticate the user in front of the rest of services through the WM. An Nginx [44] reverse proxy is also needed to provide secure connections (through HTTPS) to Keycloack as it does not support it natively.

It is worth noting that, in our current implementation, only the UA is accessible from the Internet, while the rest of the services are only accessed locally. As already mentioned, it connects with the Keycloack service to achieve user authentication via the WM. As already mentioned, we implemented UA as a web application, but the UA could be implemented as a desktop application or even a mobile one.

The database used is MySQL [45], a well-known open source relational database where metadata is stored following the ISO/IEC 23092 hierarchical structure. This database is used by WM, GCS and SS, in order to find the information associated with the files stored in the system together with the user who created them and the corresponding metadata. 

**Figure 11:**
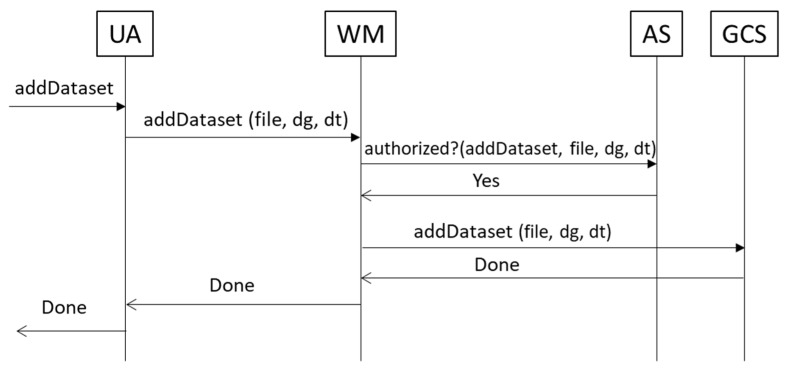
Example of operation workflow: addDataset.

WM is the entry point to GIPAMS services, in this case, from the UA. We would like to highlight the fact that UA is implemented as a web application, but any application (mobile, desktop-based, etc.) could access GIPAMS services via the WM. WM confirms user authentication by means of JWT and, depending on the operation requested by the UA (file creation, dataset group or dataset creation or modification, metadata management, etc.), it also requests authorization before calling to the corresponding service. In this way, if the operation is not authorized in the first place, the service is never invoked, minimizing unauthorized operations as soon as possible. WM communication is implemented using REST (REpresentational State Transfer) [46] endpoints. Figure 11 shows an example of the operation calls workflow between UA, WM, AS and GCS. 

AS does not require database access as it authorizes user actions based on XACML requests and rules. To do so, it uses WSO2 Balana [36], an open source implementation of XACML authorization mechanisms. The different modules request authorization for AS before genomic information (including metadata) can be accessed. The rules are stored as XML files in the ISO/IEC 23092 hierarchy as shown in Figure 10, inside dgpr or dtpr files. Apart from XACML policies and rules, these files may contain other protection information such as signatures or protection keys.

GCS manages ISO/IEC 23092 file hierarchy creation and management. The hierarchy is also stored in the database, to support searches over file structure and metadata. It also manages XML information derived from metadata and protection files. For the specific case of XML metadata files, their content is parsed and inserted in the database, so searches can be performed with SS. AS is used in order to authorize operations. Only the file creation operation does not require authorization, as it creates a new file, but the rest of the operations modifying the hierarchy (for instance, addDatasetGroup, editAnyElement or deleteAnyElement) should be authorized before they can be performed. In this case, several checks should be implemented, including file ownership.

SS provides a search interface for metadata stored inside the system database. Authorization is also required in this case, to ensure that the returned results are available for the user requesting them.

## 4. Discussion

A standards-based modular architecture such as GIPAMS has several advantages when providing security and privacy mechanisms to protect genomic information. The first one is that using standardized mechanisms favors interoperability, as implementation is based on published specifications. If there are other implementations dealing with the same standard genomic information format, GIPAMS should be able to support them. The second one is that, as each feature is implemented as an independent module, a module can be changed or updated without affecting the complete system. This provides support for new standard features in an easier way. It would even be possible to support different versions of a standard by replicating a module, as explained in Section 4.1, with GIPAMS extended architecture. 

We are currently working on the complete implementation of GIPAMS, by integrating the encryption and signature features provided by the Protection Service (PTS) with the Genomic Content Service (GCS). Moreover, we aim to implement some of the improvements proposed in this paper with modules not only based on ISO/IEC 23092 but also with GA4GH features. One implementation we plan to achieve is to combine security features from both ISO/IEC 23092 and GA4GH in the Protection Service, using protection features derived from Crypt4GH into PTS, following the path described in Section 4.1. In this way, we can achieve the integration of different standardization initiatives into GIPAMS. The next modules to implement are the Policy Service and, finally, the Reporting/Track Service modules. It is worth noting that these are not core modules for the system, but accessory ones that provide extra features to support privacy and security provisions. 

Furthermore, we also plan to account for GA4GH features when implementing new modules, i.e., to use several Genomic Content Services to support different file formats or alternatives to Authorization Service using other authorization technologies, as described further in the following section.

Finally, in Section 4.2, we describe the future work we foresee in different aspects related to GIPAMS, such as implementing different versions of the modules, search functionalities or a possible GIPAMS federation.

### 4.1. Alternatives for GIPAMS Modules Implementation Using Other Standards

GIPAMS was designed with ISO/IEC 23092 features in mind. Nevertheless, other standardized alternatives exist for some of the defined modules, as explained next.

Genomic Content Service (GCS) design is based on the ISO/IEC 23092 hierarchy. However, it is not difficult to define other GCS in order to support different genomic formats, such as Crypt4GH [5], SAM/BAM [10] or CRAM [11]. The most difficult part is the fact that these formats do not have all the features provided by ISO/IEC 23092 such as metadata, protection, hierarchy or indexing, and the connection with other modules will be more complex to achieve. 

Authorization Service (AS) could also be implemented using GA4GH Passports [6]. Nevertheless, the use of XACML rules to provide privacy can be extended to other file formats, due to XACML authorization rules and requests for flexibility. In this way, a two way interoperability mechanism could be provided, combining both format and authorization mechanisms coming from different standardization initiatives, for example, by authorizing Crypt4GH or BAM files using XACML or authorizing access to GCS using GA4GH passports. Other combinations could be also defined and implemented, especially if public API specifications are provided.

Furthermore, a Search Service (SS) could be implemented as a beacon interface [47] to perform searches of genomic information stored inside GIPAMS. This module does not currently follow any standardized mechanism. It is worth noting that the AS is contacted to check that the query is authorized. 

Policy Service (PS) relates to the AS, so the use of GA4GH Passports could be also indicated for this case. Again, XACML can be used to implement privacy rules over different genomic formats, not necessarily ISO/IEC 23092. We already have experience of the privacy protection of eHealth-related information using XACML, as described in [48,49]. Moreover, GA4GH was used to produce the Data Use Ontology (DUO) [50], which provides matching between data use restrictions on genomic data and intended research use requested by researchers. DUO could act as an alternative to XACML privacy policies when dealing with the definition of data usage restrictions. Nevertheless, an authorization mechanism is required to be as powerful as XACML currently is.

Protection Services (PTS) is currently based on the protection of information defined in ISO/IEC 23092. Again, the solution proposed for GCS, i.e., the implementation of protection methods coming from other standards, such as the ones in Crypt4GH [5], could be a feasible solution. In this case, the inclusion of encryption algorithms used in Crypt4GH in the ISO/IEC 23092 protection XML schema is a first step to extend security and protection features. On the other hand, defining more encryption algorithms into Crypt4GH data structures could also be performed. In the end, the implementation of different PTS depending on the file formats supported by GIPAMS could be also a feasible solution.

Report/Track Service (RTS) mainly derives from the need to track illegal/unusual operations. It is not really defined (at least with a formal structure and API) in ISO/IEC 23092, but it is required, as it is in other formats, as defined in [51]. The idea of defining such a module comes from MIPAMS, where a specific standard for reporting multimedia content operations was used, namely MPEG-21 Event Reporting [52].

**Figure 12:**
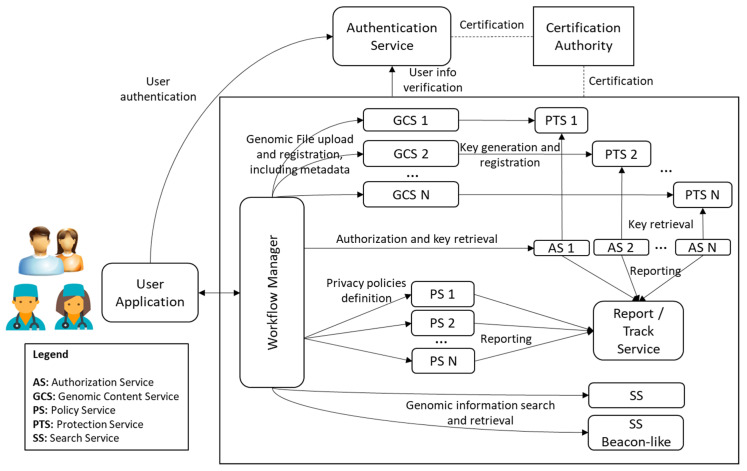
GIPAMS extended architecture.

Figure 12 shows how GIPAMS could be extended to support more standards, offering new and extended modules, providing additional functionality. Genomic Content Service (GCS) module may be replicated to support different genomic content formats. Each GCS may need a different Protection Service (PTS), as shown in Figure 12, depending on the file format and its security and protection mechanisms. Furthermore, Policy and Authorization Services (PS, AS) modules may be replicated to support different authorization mechanisms coming, for example, from ISO/IEC 23,092 or GA4GH. Finally, the Search Service (SS) could be implemented using the metadata stored in the database, as it is now, or by providing a Beacon-such as [47] SS. 

### 4.2. Future Work

After completing GIPAMS implementation, we will integrate other standardization initiatives as alternate versions of the modules, as shown in Figure 12 This will lead us to GIPAMS version 2, where different genomic formats and related standards may interoperate. The complete implementation of PTS integrating ISO/IEC 23092 and the GA4GH mechanisms provides proof of concept that standards interoperability can really be achieved. We have already performed some testing on that direction, so we are confident that we will achieve complete PTS soon.

Furthermore, several GIPAMS instances could be established at different locations, providing a federated system. The definition of global access rules over metadata stored at the different locations may allow for the provision of a federated search whilst guaranteeing the privacy and security of the results. Having such a system, that is, GIPAMS federation, provides several advantages. The first is that each location only manages their own genomic information, so less storage and transmission is required. This is better than having several copies of the complete system. The second one is that although each location only contains its own metadata, the federated metadata search provides each location with the possibility of accessing the complete metadata describing other genomic studies that may be relevant. Discovering relevant information through the federated search may provide researchers with the opportunity requesting request access to genomic data (or part of it) in a secure and controlled way, as they may find genomic studies of interest in an easier way. In any case, rules for metadata should be checked before returning the results, as is already performed in one GIPAMS. Once a GIPAMS federation is established, new services and opportunities for researchers may arise, based on the federated search.

In order to facilitate relevant genomic information discovery, a metadata search may also include information on whether the genomic data associated with some metadata can be accessed partially or completely and how this access can be requested. This can be controlled by means of the access rules defined for genomic metadata and data and of course supported by the encryption/decryption of the information. This could be very useful for rare diseases, where a few cases are available and the privacy and security standards should be maximal in order to not to reveal patient identity, but, at the same time, provide the maximum visibility to find a treatment thanks to research results.

Finally, the application of different security mechanisms provides privacy protection for the genomic information managed inside GIPAMS. First of all, the communication between the user application and the rest of the modules/services is conducted through a secure channel. Moreover, tracking of user actions and unique user identification are also implemented. For the protection of the genomic data and metadata, different encryption techniques can be applied. To control which actions users can perform in the system, privacy protection rules can be defined with a high level of granularity. Although information could be leaked from the user’s application, we should be aware of two relevant facts: (1) users are identified and “trustable” and (2) all actions related to the information are tracked.

## 5. Conclusions

This paper presents how we can provide security and privacy to genomic information using standards in a modular architecture. This is the basic idea of GIPAMS, a modular architecture for the secure management of genomic information, as introduced in Section 2.

For its purpose, we mainly focused on ISO/IEC 23092 features [2,3], as this is a genomic information format which has been developed using privacy by design principles since its inception. Such features, among others, are as follows: protection of information and privacy rules associated with genomic information, including metadata, a hierarchical structuring of the information, starting from the complete file and finishing on the blocks containing genomic information itself, and the possibility of associating security information with a high level of granularity.

Taking these features as a starting point, we developed a first implementation of GIPAMS, as a continuation of previous preliminary work [1].

Therefore, inside GIPAMS, we used the ISO/IEC 23092 hierarchical structure as a base point to facilitate the integration of other existing genomic information formats. In this way, search and linkage between different genomic formats could be easily achieved with the implementation of differentiated modules for each genomic content format. The Workflow Manager (WM) may orchestrate calls to the corresponding modules. Related to this, the extension metadata mechanism defined in ISO/IEC 23092 also assists in the inclusion and integration of new metadata, facilitating the implementation of more specific and accurate searches, thereby providing access to more research results with a common interface.

The idea of describing a modular architecture for managing content comes from MIPAMS [31], which was defined for the secure management of multimedia content. Some of the modules have evolved, but the underlying ideas remain, i.e., the provision of different modules in charge of the content creation and management, protection, governance and access control (by means of licenses) of multimedia content.

Therefore, by combining MIPAMS with ISO/IEC 23092, we defined GIPAMS module functionalities, how the communication among them should be performed and ways in which to implement a first version. In this paper, we went a step forward, as we did not only use ISO/IEC 23092 to support genomic information, but we also included GA4GH security mechanisms and authentication features, as explained before.

To integrate GA4GH standards into GIPAMS, we firstly identified the initiatives that can be included in current (or an evolved version of) GIPAMS modules. One of them is Crypt4GH [5], as it defines a file encryption standard and it involves both GCS and PTS modules. Another GA4GH initiative identified is Passports [6,7] and Authentication and Authorization Infrastructure (AAI) [8,9]. Both of them could be integrated or used as an alternative to the AS module. There are other initiatives inside GA4GH, such as Phenopackets [53,54], that we are also considering for its inclusion and support as part of GIPAMS. It is worth noting that Phenopackets is also an ISO standard [54].

## Figures and Tables

**Table 1:** EGA metadata mapping.

MPEG-G Field	EGA Field
Title	Study-STUDY_TITLE
Type	Study-STUDY_TYPE
Abstract	Study-STUDY_ABSTRACT
ProjectCentre	Study-CENTER_PROJECT_NAME
Description	Study-STUDY_DESCRIPTION
Sample-TaxonId	Assembly-TAXON_ID
Sample-Title	Assembly-TITLE

**Table 2:** NCBI metadata mapping.

MPEG-G Field	NCBI Field
Title	BioProject-Title
Type	BioProject-ProjectTypeSubmission
Abstract	Non existent
ProjectCentre	BioProject-Organization
Description	BioProject-Description
Sample-TaxonId	BioSample-TAXON_ID
Sample-Title	BioSample-TITLE

**Table 3:** Attribute extension fields.

Attribute Extension	NCBI Field
StudyDesign	BioSample-Attribute-study design
BodySite	BioSample-Attribute-body site
AnalyteType	BioSample-Attribute-analyte type
IsTumor	BioSample-Attribute-is tumor

## Data Availability

Not applicable.
